# Mechanisms and proteins involved in long-distance interactions

**DOI:** 10.3389/fgene.2014.00028

**Published:** 2014-02-18

**Authors:** Oksana Maksimenko, Pavel Georgiev

**Affiliations:** ^1^Laboratory of Gene Expression Regulation in Development, Institute of Gene Biology, Russian Academy of SciencesMoscow, Russia; ^2^Department of the Control of Genetic Processes, Institute of Gene Biology, Russian Academy of SciencesMoscow, Russia

**Keywords:** chromatin looping, long-range interactions, chromatin insulators, *cis*-regulation, transcription, enhancers

## Abstract

Due to advances in genome-wide technologies, consistent distant interactions within chromosomes of higher eukaryotes have been revealed. In particular, it has been shown that enhancers can specifically and directly interact with promoters by looping out intervening sequences, which can be up to several hundred kilobases long. This review is focused on transcription factors that are supposed to be involved in long-range interactions. Available data are in agreement with the model that several known transcription factors and insulator proteins belong to an abundant but poorly studied class of proteins that are responsible for chromosomal architecture.

## INTRODUCTION

In recent years, considerable progress has been made in understanding chromosome organization (for reviews, see Gibcus and Dekker, 2013;Krijger and de Laat, 2013; Nora et al., 2013; Tanay and Cavalli, 2013). High-resolution chromosome conformation capture techniques have provided evidence that chromosomes in the genomes of human, mouse, and *Drosophila* are partitioned into a series of discrete topologically associating domains (TADs; Lieberman-Aiden et al., 2009; Dixon et al., 2012; Nora et al., 2012; Sexton et al., 2012). Their characteristic feature is that regulatory elements within a TAD display extensive long-range interactions with each other but interact far less frequently with regulatory elements located outside their domain. The size of TADs ranges from 10 to 500 kb, with a median of about 100 kb, in *Drosophila* (Sexton et al., 2012) and from slightly less than 100 kb to several megabases, with a median of 1 Mb, in humans and mice (Dixon et al., 2012; Nora et al., 2012). Within a TAD, numerous local chromatin loops are formed between enhancers, silencers, and promoters, with their length in some cases exceeding 100 kb (Li et al., 2012; Sanyal et al., 2012; Shen et al., 2012; Phillips-Cremins et al., 2013).

Two decades have elapsed since insulators were recognized as a specific class of DNA sequence elements that contribute to organization of independent gene function domains by restricting the enhancer and silencer functions (Ghirlando et al., 2012; Herold et al., 2012; Kirkland et al., 2013). However, although considerable progress has been made in the study of chromosomal architecture, we still do not have a clear mechanistic picture of how long-range interactions between distant regulatory regions are established and maintained through the cell cycle. In the past few years, a concept has been formed that there is a special class of architectural proteins, including some known insulator proteins, that are responsible for global chromosome architecture as well as for local regulation of enhancer–promoter interactions (Maksimenko et al., 2008; Holwerda and de Laat, 2012; Gibcus and Dekker, 2013; Nora et al., 2013; Kyrchanova and Georgiev, 2014). This paper is an attempt to summarize recent progress in understanding the role (function) of transcription factors and insulator proteins as architectural proteins.

## *DROSOPHILA* TRANSCRIPTION FACTORS THAT ARE INVOLVED IN DISTANT ENHANCER–PROMOTER INTERACTIONS

*Drosophila* is a unique model system to study long-distance interactions between regulatory elements. Using transposon-mediated transformation or attP-phage-based integration and manipulation with recombination systems, it is possible to obtain different combinations of the regulatory elements in the same genomic position in order to study the role of particular regulatory elements in reporter expression (for reviews, see Bischof et al., 2007; Venken and Bellen, 2012). These approaches have made it possible to discover several “tethering” elements near promoters that ensure specific long-distance interactions between enhancers and corresponding promoters (Calhoun et al., 2002; Calhoun and Levine, 2003; Akbari et al., 2008; Melnikova et al., 2008). However, the proteins that bind to tethering elements and are responsible for their activity have not yet been identified.

**FIGURE 1:**
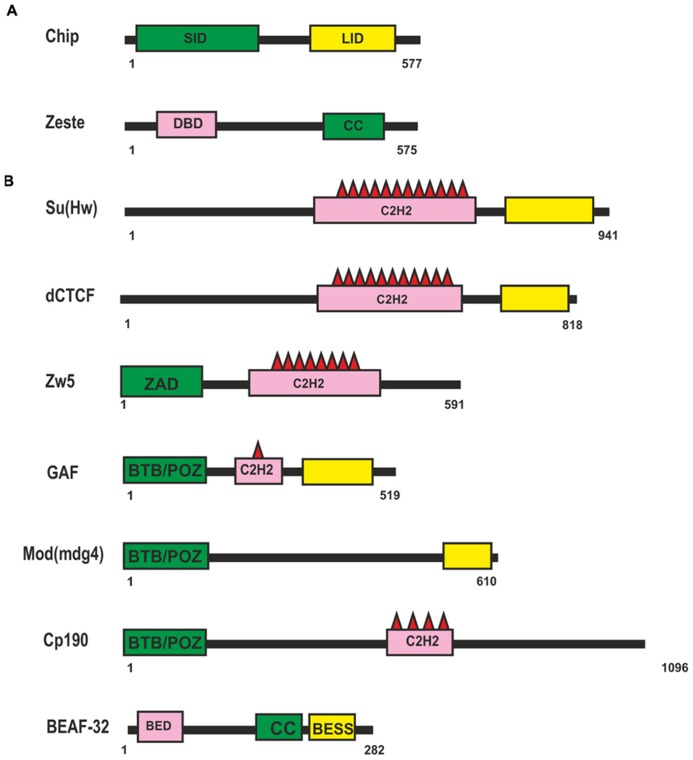
***Drosophila* proteins suggesting to be involved long-distance interactions.** **(A)** Domain organization of the Chip and Zeste proteins. **(B)** Domain organization of the *Drosophila* insulator proteins. Self-interaction, protein-protein interaction and DNA-binding domains are shown as green, yellow, and pink boxes respectively. Abbreviations: self-interaction domain (SID), LIM-interaction domain (LID), DNA binding domain (DBD), coiled-coil domain (CC), Zinc-fingers of C2H2 type (C2H2), Zinc-finger Associated Domain (ZAD), BTB/POZ domain, BED-type (BEAF and DREF) zinc finger domain (BED), BEAF, Su(var)3-7, and Stonewall domain (BESS).

To date, only two known transcription factors, Chip and Zeste, have been considered tobe involved in supporting distant enhancer–promoter interactions in *Drosophila* (**Figure 1A**). The Chip protein can form dimmers and mediate interactions between different classes of transcription factors (for review, see Matthews and Visvader, 2003). Chip has two domains well-conserved among higher eukaryotes: an amino-terminal homodimerization domain (SID) and a carboxy-terminal LIM interaction domain (LID; **Figure 1A**). The LID domain interacts with LIM-homeodomain (LIM-HD) and LIM-only (LMO) proteins, which have important roles in cell fate determination, tissue development, and cytoskeletal organization. Recent data show that Chip is responsible for cooperative binding of LIM-HD and GATA proteins onto target promoters and enhancers (Heitzler et al., 2003; Bronstein et al., 2010). There is indirect genetic evidence that Chip supports long-range enhancer–promoter interactions in the *cut* locus (Morcillo et al., 1997) and *achaete-scute* complex (Ramain et al., 2000; Heitzler et al., 2003).

A putative role of another protein, Zeste (**Figure 1A**), in distant interactions has also been evidenced only in genetic studies with transgenic lines (Qian et al., 1992; Laney and Biggin, 1997; Kostyuchenko et al., 2009). Zeste is a sequence-specific DNA-binding protein that binds to the regulatory regions of many genes, including the *white* and *Ubx* genes, and stimulates their expression (Benson and Pirrotta, 1988; Chen and Pirrotta, 1993). A unique feature of Zeste is that it binds cooperatively to multiple binding sites as a higher-order homo-oligomer (Chen and Pirrotta, 1993). Zeste oligomerization is the result of interactions mediated by carboxy-terminal leucine zipper motifs. In particular, Zeste binds to the enhancer and promoter of the *white* gene (Qian et al., 1992). In transgenic lines, Zeste is strongly required for the distant interaction between the eye enhancer and the *white* promoter across the heterologous *yellow* gene (Kostyuchenko et al., 2009). At the same time, inactivation of Zeste has no effect on the activity of the eye enhancer when it is located relatively close to the *white* promoter. The deletion of Zeste binding sites in the upstream promoter region does not affect the basal level of *white* expression but eliminates Zeste-dependent long-range communication between the enhancer and the promoter. Thus, it appears that Zeste is not required for basal activity of the promoter but contributes to organization of specific enhancer–promoter interactions. However, there is no direct evidence that Zeste itself is sufficient for establishing enhancer–promoter interactions. Therefore, it may well be that additional, as yet unknown transcription factors cooperate with Zeste to support specific enhancer–promoter interactions stimulating *white * expression.

## *DROSOPHILA* INSULATOR PROTEINS ARE LIKELY CANDIDATES FOR ARCHITECTURAL TRANSCRIPTION FACTORS

Most information about potential transcription factors involved in long-range interactions has been obtained in studies on *Drosophila* insulators. The *Drosophila* genome contains many sequences with an insulator function (Herold et al., 2012). The first insulators to be identified were scs and scs’ located at the boundaries of two heat shock 70 genes (Kellum and Schedl, 1991, 1992). Two proteins, Zw5 and BEAF (**Figure 1B**), bind to scs and scs’, respectively, and partially account for their insulator properties (Zhao et al., 1995; Gaszner et al., 1999). The best characterized insulator consisting of reiterated binding sites for the Su(Hw) protein (**Figure 1B**) was found in the regulatory region of the *gypsy* retrotransposon (Holdridge and Dorsett, 1991; Geyer and Corces, 1992). The Su(Hw) protein associates with thousands of genomic sites, with the vast majority of them carrying a single copy of the corresponding sequence (Golovnin et al., 2003; Parnell et al., 2003; Kuhn-Parnell et al., 2008; Soshnev et al., 2012, 2013).

Insulators named Mcp, Fab-6, Fab-7, and Fab-8 were identified at the boundaries of enhancer domains regulating proper activation of the *Abd-B* gene in the *Bithorax* complex (Gyurkovics et al., 1990; Barges et al., 2000; Hogga et al., 2001; Schweinsberg et al., 2004; Gruzdeva et al., 2005; Rodin et al., 2007; Iampietro et al., 2008, 2010; Aoki et al., 2012). Binding sites for a *Drosophila* homolog of vertebrate insulator protein CTCF (dCTCF; **Figure 1B**) were found in Mcp, Fab-6, and Fab-8 insulators (Moon et al., 2005; Holohan et al., 2007). Other transcription factors – GAF, ELBA, and BEAF-32 – were also found to frequently bind to known *Drosophila* insulators. In addition, several insulators were described for which DNA binding proteins have not yet been identified (Herold et al., 2012).

**FIGURE 2:**
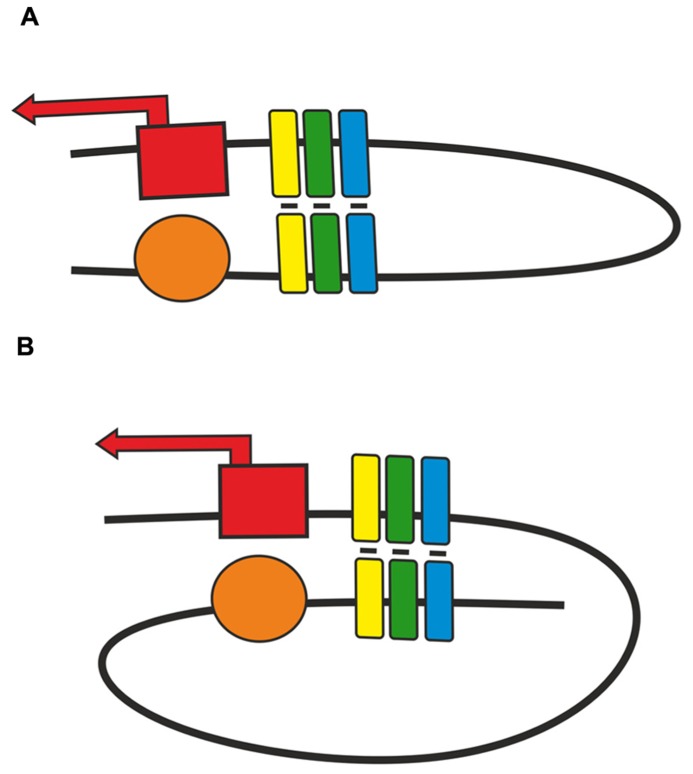
**Two modes of pairing between two copies of an insulator inserted in either **(A)** opposite or **(B)** same orientation.** Presumptive proteins responsible for insulator pairing are shown as a cluster of yellow, green, blue boxes. Red box with solid arrow indicates promoter region, orange oval – enhancer element.

As shown in transgenic lines, pairing of two identical insulators can support distant activation of a promoter by an enhancer or yeast GAL4 activator (Cai and Shen, 2001; Muravyova et al., 2001; Kyrchanova et al., 2007; Kyrchanova et al., 2008a, b). The relative orientation of two identical insulators defines the mode of loop formation that either allows or blocks enhancer (GAL4)–promoter interaction (Kyrchanova et al., 2008a,b). This phenomenon is explained by the assumption that when the insulators are located in opposite orientations, the loop configuration is favorable for communication between regulatory elements located beyond the loop (**Figure 2**). The loop formed by two insulators located in the same orientation juxtaposes two elements located within and beyond the loop. Supposedly, this orientation-dependent interaction is accounted for by at least two insulator-bound proteins that are involved in specific protein–protein interactions.

It has also been found that two identical insulators can support interactions between regulatory elements located in transgenes inserted at distances up to several megabases from each other (Sigrist and Pirrotta, 1997; Muller et al., 1999; Kravchenko et al., 2005; Vazquez et al., 2006; Li et al., 2011, 2013). The most striking example is the insulator termed Homie that is located between the *TER94* promoter and regulatory region of the *eve *gene (Fujioka et al., 2009). The presence of Homie in a transgene as far as 3.3 Mb away from the endogenous copy facilitates long-range communication between endogenous *eve* enhancers located near Homie and a promoter placed on the transgene (Fujioka et al., 2009, 2013). These facts suggest that proteins bound to insulators can support very specific distant interactions through the cell cycle. Thus, insulators are good candidates to form interactive boundaries partitioning *Drosophila* chromosomes into TADs. Indeed, insulator-bound proteins are frequently found at the presumed borders of TADs (Sexton et al., 2012).

To support specific long-range interactions, insulator proteins should have homodimerization domains. Three insulator proteins – Su(Hw), Zw5, and dCTCF (**Figure 1B**) – contain multiple C2H2 zinc fingers (Kim et al., 1996; Gaszner et al., 1999; Moon et al., 2005). To date, these proteins have not been examined for the presence of dimerization domains. Only Zw5 was found to contain a zinc finger-associated domain (ZAD) specific for insects at the N-terminus (Gaszner et al., 1999; Blanton et al., 2003). More than 90 ZAD-proteins were also identified in the *Drosophila* genome (Chung et al., 2002), but they have not yet been studied sufficiently. They are characterized by a conserved constellation of four cysteines within the ZAD, which form a zinc-coordinated fold. The crystal structure of the ZAD of Grauzone protein provides evidence that two ZAD molecules interact in a head-to-tail mode to form a dimer, which suggests that ZAD domains of other proteins are also able to self-associate (Jauch et al., 2003). Therefore, the ZAD domain of Zw5 may be involved in distant interactions, but this assumption requires experimental verification.

Moreover, it has been shown that Su(Hw) interacts with the CP190 protein and Mod(mdg4) isoform named Mod(mdg4)-67.2 (Büchner et al., 2000; Gause et al., 2001; Ghosh et al., 2001; Pai et al., 2004; Golovnin et al., 2007), and the dCTCF protein interacts with CP190 (Gerasimova et al., 2007; Mohan et al., 2007).

The GAF, Mod(mdg4)-67.2, and CP190 proteins have the BTB (bric-a-brac, tramtrack, and broad complex)/POZ (poxvirus and zinc finger) domain at the N-terminus. The BTB is a conserved protein–protein interaction motif contained in a variety of transcription factors involved in development, chromatin remodeling, insulator activity, and carcinogenesis (Stogios et al., 2005; Perez-Torrado et al., 2006). All well-studied mammalian BTB domains form obligate homodimers and, rarely, tetramers. The BTB domains of *Drosophila* GAF and Mod(mdg4)-67.2 factors belong to the “ttk group,” which contains several highly conserved sequences not found in other BTB domains, and exist as higher-order multimers (Zollman et al., 1994; Espinas et al., 1999; Mahmoudi et al., 2002; Bonchuk et al., 2011).

The role of BTB domains and especially of GAF and Mod(mdg4)-67.2 in organization of long-distance interactions either between insulators or between an enhancer and a promoter have been discussed for a long time. Electron microscopic and DNA pull-down experiments have shown that GAF complexes can form a protein link between separate DNA elements *in vitro* (Katsani et al., 1999; Mahmoudi et al., 2002). Similar results have also been obtained for the Bach1 BTB/POZ protein interaction domain required for the formation of looped DNA structures between different regulatory elements within the human β-globin LCR, as visualized by atomic force microscopy *in vitro* (Yoshida et al., 1999). As shown by functional *in vivo* assays, GAF can facilitate gene activation in a heterologous model system such as human 911 cells (Mahmoudi et al., 2002) and yeast (Petrascheck et al., 2005) by acting as an anchor that links the remote GAL4 binding sites to the promoters. However, binding sites for GAF do not support distant interaction between GAL4 activator and the *white* promoter in *Drosophila* transgenic lines (Bonchuk et al., 2011), while binding sites for Zw5, dCTCF, or Su(Hw) can support such interactions in the same model system (Kyrchanova et al., 2008a). Thus, there is no conclusive experimental evidence for the ability of GAF to support long-distance interactions in *Drosophila*. On the other hand, oligomerization of the BTB domains is required for cooperative binding of GAF to many adjacent sites in the same regulatory region (enhancer, insulator, or promoter; Katsani et al., 1999). As a result, GAF can open chromatin regions, thereby allowing the recruitment of other transcription factors to regulatory regions (Leibovitch et al., 2002). A similar role may be played by the self-association domain located at the C-terminus of BEAF protein (Hart et al., 1997; Gilbert et al., 2006).

Biochemical experiments have shown that each BEAF protein (**Figure 1B**) binds with its N-terminal BED finger domain to specific DNA motif CGATA, while BEAF trimers bind with high affinity to clusters of CGATA motifs (Hart et al., 1997). According to the results of genome-wide analysis, BEAF preferentially binds to such clusters in the promoter regions of active genes and is required for stimulation of their transcription (Emberly et al., 2008; Jiang et al., 2009).

A new insulator complex, named ELBA, recently described in *Drosophila* (Aoki et al., 2012) is composed of two proteins, Elba1 and Elba2, which share a conserved C-terminal “BEN domain” mediating binding to DNA. The third protein, Elba3, is responsible for “dimerization” of the Elba1-2 BEN domains and is encoded by the gene closely linked to Elba1. In this case, dimerization domain is required for cooperative binding of two BEN domains to corresponding insulator sites. Thus, dimerization domains in many insulator-bound proteins may be essential for effective binding of insulator proteins to chromatin but not for organization of long-distance interactions.

The Su(Hw), Mod(mdg4)-67.2, and CP190 proteins colocalize in discrete foci, named insulator bodies, in the *Drosophila* interphase cell nucleus (Gerasimova et al., 2000; Pai et al., 2004). Hence, it has been asserted (Gerasimova et al., 2000) that the insulator bodies arise via association of individual Su(Hw)-containing nucleoprotein complexes located at distant chromosomal sites. Hypothetically, a number of Su(Hw) insulators coalesce into an insulator body owing to interactions between the BTB domains of insulator proteins Mod(mdg4)-67.2 and CP190. However, recent results show that the insulator bodies are aggregates of insulator proteins that resemble well-known promyelocytic leukemia nuclear bodies (PML-NB) and stress bodies, which comprise many unrelated proteins (Golovnin et al., 2008, 2012; Schoborg et al., 2013). Thus, there is no direct evidence that Mod(mdg4) and CP190 are important for supporting interactions between insulators located at a large distance from each other. Since inactivation of either CP190 or Mod(mdg4)-67.2 leads to weaker Su(Hw) binding to chromosomes (Pai et al., 2004; Golovnin et al., 2007; Schwartz et al., 2012), it seems likely that the BTB-containing proteins are important for cooperative binding of insulator proteins to their sites and consequent formation of insulator complexes.

In conclusion, it should be noted that some of *Drosophila* insulator proteins are good candidates to be architectural proteins. However, the mechanisms of and possible protein domains involved in long-distance interactions have not yet been identified.

## COOPERATION OF CTCF WITH COHESIN IN CHROMATIN ARCHITECTURE OF MAMMALIAN GENOME

CTCF (CCCTC-binding factor) is regarded as the main insulator protein in mammals (Ohlsson et al., 2010; Chaumeil and Skok, 2012; Lee and Iyer, 2012; Merkenschlager and Odom, 2013). This protein is ubiquitously expressed across most mammalian tissues (Wendt et al., 2008) and is required for early mouse development (Fedoriw et al., 2004), participating in cell-cycle progression, apoptosis, and cell differentiation (Splinter et al., 2006; Heath et al., 2008; Soshnikova et al., 2010). Many independent experiments on genome-scale mapping of CTCF binding in cells of different mammalian tissues have revealed its preferential binding at the gene-dense regions but with little or no enrichment in promoters (Kim et al., 2007; Chen et al., 2008; Wang et al., 2012; Lee et al., 2012). This protein localizes at the DNase I-hypersensitive sites, open chromatin determinants that are generally common across cell types (Song et al., 2011). There is ample experimental evidence for the role of CTCF in organization of chromatin architecture in particular loci and formation of TADs (Chaumeil and Skok, 2012; Herold et al., 2012; Holwerda and de Laat, 2012; Merkenschlager and Odom, 2013).

**FIGURE 3:**
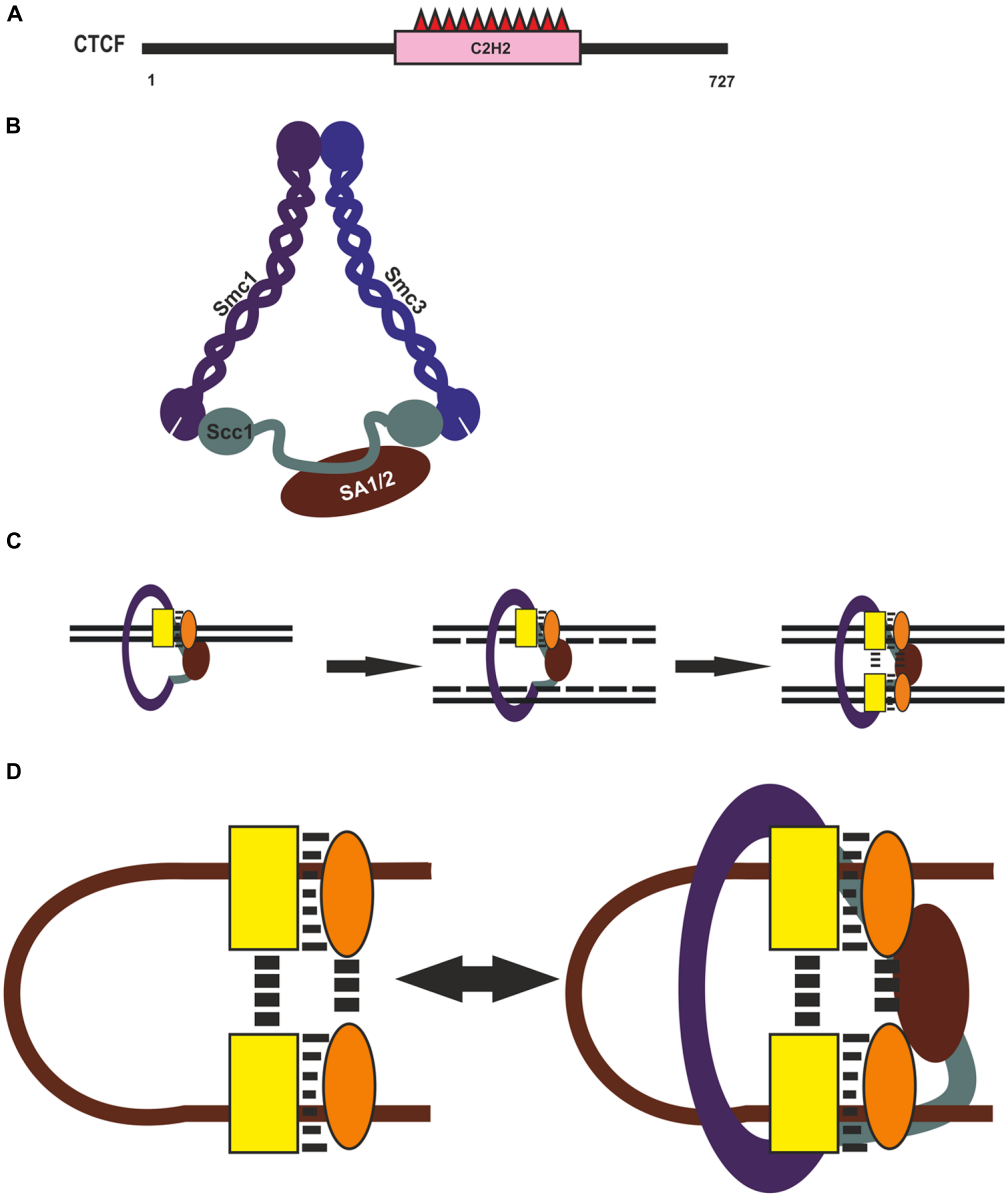
**CTCF and cohesin complex in chromatin architecture.** **(A)** Domain organization of CTCF. **(B)** Cohesin complex. Structural Maintenance of Chromosomes (Smc), Sister Chromatid Cohesion (Scc), Stromal Antigen (SA). **(C)** Possible role of Cohesin in reproducing of CTCF complex on newly synthesized DNA during replication. CTCF and assumptive interaction protein are shown by yellow rectangle and orange oval. **(D)** Diagram illustrating possible role of CTCF with its partners and Cohesin in organization and supporting of long-distance interactions.

Although CTCF is recognized as the main architectural protein, information on the involvement of its domains in long-distance interactions is scarce (**Figure 3A**). The central part of its molecule contains 11 C2H2 zinc fingers (ZFs), with ZFs 4–7 recognizing the core consensus DNA motif (Nakahashi et al., 2013). Nonconserved flanking DNA sequences are recognized by ZFs 1–2 and ZFs 8–11 clusters, which also stabilize CTCF. This protein is capable of self-association, but domains involved in this process have not been characterized sufficiently (Yusufzai and Felsenfeld, 2004). Pant et al. (2004) obtained evidence for a pairwise interaction between the C-terminal end of one CTCF molecule and the ZF domain of another *in vitro*. However, the ZF domain of CTCF can also interact with many different proteins, including CHD8, Sin3A, and YB-1 (Chernukhin et al., 2000; Lutz et al., 2000; Ishihara et al., 2006). Therefore, such a ZF-mediated mechanism is unlikely to account for specific long-distance interactions between CTCF binding sites, and further studies are needed to identify CTCF domains responsible for such interactions.

An important role for CTCF-mediated distant interactions has been suggested for the cohesin complex (Holwerda and de Laat, 2012; Lee and Iyer, 2012; Mehta et al., 2013; Merkenschlager and Odom, 2013). This macromolecular complex, conserved from yeast to human, is responsible for the fidelity of chromosome segregation during mitosis and meiosis, keeping the sister chromatids together from S phase to anaphase (for review, see Peters, 2012; Dorsett and Merkenschlager, 2013; Remeseiro and Losada, 2013). The complex is composed of four protein components: two long coiled-coil molecules, Smc1 and Smc3, which form an open-ended heterodimer; Scc1 (Rad21), which bridges its open end; and SA1 (or SA2), which interacts with Scc1 and is external to the Smc1/Smc3/Scc1 trimer (**Figure 3B**). Thus, the three core subunits of cohesin – Smc1, Smc3, and Scc1 (Rad21) – form a ring-shaped structure, and the SA1 and SA2 proteins interact with it in a mutually exclusive manner. It has been proposed that such complexes mediate chromatid cohesion by trapping the two sister DNA molecules inside the cohesin ring and can have an effect on chromatin structure, forming or stabilizing intrachromatid loops (**Figure 3C**).

The results of genome-wide analysis of CTCF and cohesin binding show that their patterns largely overlap (Parelho et al., 2008; Wendt et al., 2008). CTCF and subunits of the cohesin complex coprecipitate in the nuclear lysate, and SA2 directly interacts with CTCF *in vitro* (Xiao et al., 2011), suggesting that SA2 may be responsible for cohesin recruitment to CTCF-binding sites. Another potential participant in the stabilization of CTCF–cohesin interaction is the DEAD-box RNA helicase p68, which functions in association with the steroid receptor RNA activator (SRA; Yao et al., 2010). As shown by genome-wide ChIP-Seq analysis, 22% of p68 peaks are associated with CTCF-binding sites, and 7% of CTCF sites bind p68. The p68/SRA complex interacts with both CTCF and cohesin, and depletion of p68 or SRA results in the loss of cohesin binding to CTCF. On this basis, the authors (Yao et al., 2010) suggest that RNA helicase stabilizes the cohesin–CTCF interaction.

In CTCF-depleted cells, only a small part of cohesin sites is lost, indicating that CTCF is only one of many factors recruiting the cohesin complex to chromatin (Wendt et al., 2008; Hadjur et al., 2009; Nativio et al., 2009). For example, it has been shown that transcription factor Klf4 interacts with the cohesin complex and recruits it to the Oct4 distal enhancer (Wei et al., 2013). A CTCF-independent role for cohesin in transcription regulation was also demonstrated by Schmidt et al. (2010), who revealed cohesin and estrogen receptor co-binding near upregulated genes upon estrogen treatment of MCF-7 cells. Of interest are recent data that cohesin and CTCF contribute differentially to the topological domain architecture (Zuin et al., 2013), which further support the model that many additional transcriptional factors in cooperation with cohesin are involved in organization of long-distance interactions.

Recently cohesin binding has been revealed at most of active regulatory regions (Schaaf et al., 2013; Yan et al., 2013), suggesting that cohesin can support but not organize specific long-distance interactions between CTCF binding sites (**Figure 3D**). Genome-wide studies have shown that only a minor part of CTCF binding sites are involved in loop formation, which is evidence that additional proteins may participate in this process (Handoko et al., 2011; Dixon et al., 2012; Sanyal et al., 2012). Many CTCF-binding partners that can aid in the diverse functions of CTCF have been reported to date (for reviews, see Herold et al., 2012; Lee and Iyer, 2012). For example, zinc-finger protein Prdm5 interacts with CTCF and colocalizes with it at many genomic sites (Galli et al., 2013). Taken together, these observations suggest that CTCF helps in recruiting additional transcriptional factors that, in turn, might be involved in mediating in specific selective long-distance interactions between CTCF binding sites (**Figure 3D**).

## COOPERATION OF MEDIATOR AND COHESIN IN SHORT-RANGE ENHANCER–PROMOTER INTERACTIONS IN MAMMALS

Cohesin copurifies and colocalizes with the Mediator complex, which binds to most of active promoters and enhancers in eukaryotes (Ebmeier and Taatjes, 2010; Kagey et al., 2010). Mediator is a highly conserved, large multisubunit complex comprising 25 subunits in yeast and 30 or more subunits in higher organisms (for reviews, see Malik and Roeder, 2010; Ansari and Morse, 2013). Several Mediator subunits have been shown to interact with various activators both in yeast and metazoans (Brzovic et al., 2011; Vojnic et al., 2011), with its specific subunits interacting with Pol II subunits and other general transcription factors bound to promoters (Takagi et al., 2006; Esnault et al., 2008; Cai et al., 2010). The classical model suggests that Mediator acts as an adaptor that conveys transcription signals from activators to the general transcription machinery to help initiate transcription by Pol II (Malik and Roeder, 2010; Ansari and Morse, 2013).

It has been shown that DNA looping takes place between enhancers and promoters occupied by the Mediator and cohesin complexes (Kagey et al., 2010; Seitan et al., 2011). Inactivation of cohesin or Mediator components leads to partial loss of enhancer–promoter interactions. On this basis, it has been suggested that Mediator and cohesin together bridge cell-type-specific enhancer–promoter interactions (Phillips-Cremins et al., 2013). The model proposed by these authors is also based on the ability of the large Mediator complex to simultaneously interact with enhancer-bound activators and general transcription factors bound to a promoter. Hence, Mediator may potentially bring together remote enhancers and promoters, while the cohesin complex stabilizes such interactions by forming a ring around an enhancer and a promoter sites. This model is attractive, but it has not yet been supported by direct experimental evidence. On the contrary, there is at least one example demonstrating that the loss of a cohesin-associated site at the one of Myc-mediated enhancers does not lead to the loss of another paired site on the interacting promoter (Yan et al., 2013).

Cohesin also extensively colocalizes with transcription activators (Yan et al., 2013) and Polycomb repressive complex 1 (Schaaf et al., 2013), facilitating the recruitment of these proteins to their sites. Moreover, cohesin is essential for protein complex formation on newly synthesized DNA during replication, since it is responsible for holding the nascent sister chromatids together at regulatory regions (Yan et al., 2013). Such a role of cohesin binding in promoting re-establishment of transcription factors on corresponding regulatory elements during the cell cycle suggest the ability for cohesin to help in recruiting CTCF and some unknown architectural proteins onto newly synthesized DNA during replication (**Figure 3C**). These proteins organize specific long-distance interactions, which, in turn, are also stabilized with participation of cohesin (**Figure 3D**). In accordance with this assumption, cohesin depletion in non-cycling mouse thymocytes proved to have no significant effect on preexisting architectural compartments but diminished interactions between some cohesin-bound sites (Seitan et al., 2013).

## INSULATOR–PROMOTER INTERACTIONS IN VERTEBRATES AND *DROSOPHILA*

As shown in several recent studies, CTCF binding sites frequently interact with active promoters, and CTCF may be involved in organization of enhancer–promoter interactions (Handoko et al., 2011; Sanyal et al., 2012). The molecular mechanism of the CTCF–promoter interaction might be explained by the recent finding that CTCF interacts with TAF3, a component of the basal TFIID transcriptional machinery (Liu et al., 2011). In *Drosophila*, the enhancer-blocking activity of several promoters and insulators depends on general transcription factors that inhibit RNAP II elongation (Chopra et al., 2009). It has been speculated that insulators interact with components of the RNAP II complex at stalled promoters and that the resulting chromatin loops can prevent the inappropriate activation of stalled genes by enhancers associated with the neighboring locus. *Drosophila* insulators located on the 3′-side of genes interact with promoters, and these interactions are in some cases necessary for the basal activity of the promoters (Erokhin et al., 2011; Kyrchanova et al., 2013). In addition to the possible role of a gene loop in the enhancement of RNAP II recycling and mRNA export, insulators may serve to bring to the promoter the remodeling and histone modification complexes that improve the binding and stabilization of the TFIID complex. In transgenic lines, insulators proved to interact with different promoters, suggesting that insulator proteins can interact with components of general transcription complex assembled on promoters. Insulator protein GAF interacts with TAF3, as does human CTCF (Chopra et al., 2008), which indicates that TAF3 may be a key protein in the TFIID complex that is responsible for nonspecific interaction between insulators and promoters.

## TRANSCRIPTION FACTORS INVOLVED IN ERYTHROPOIESIS AS POSSIBLE ORGANIZERS OF ENHANCER–PROMOTER INTERACTIONS

Current knowledge of vertebrate proteins maintaining chromatin loops between enhancers and promoters has come mainly from studies on genes involved in erythropoiesis, the process dependent on lineage-specific transcription factors GATA1, GATA2, Tal1, E2A, FOG, and Klf1 (for review, see Cantor and Orkin, 2002; Anantharaman et al., 2011; Palstra and Grosveld, 2012).

**FIGURE 4:**
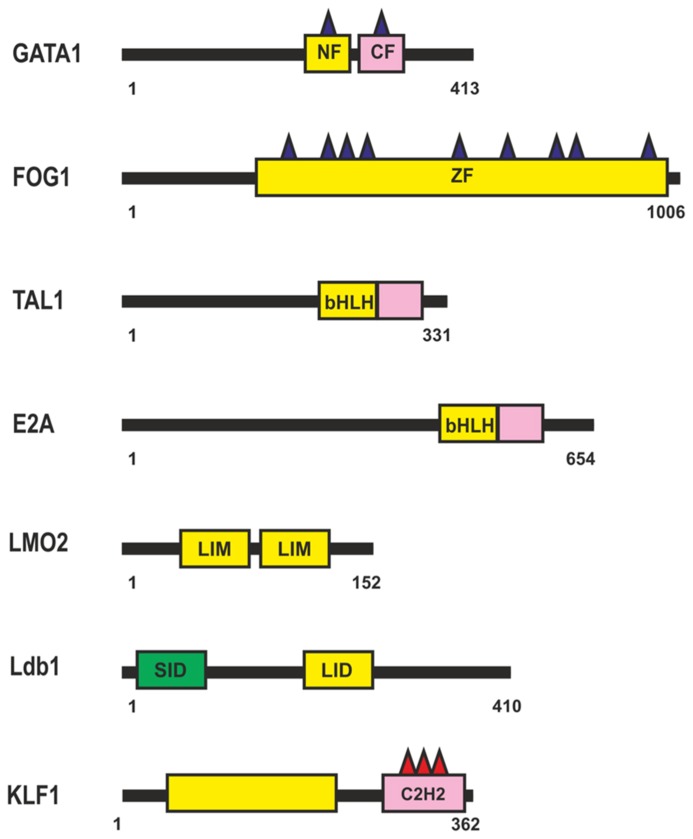
**Transcription factors involved in regulation of the β-globin locus during erythropoiesis.** N-termini C4-type zinc finger domain (NF), C-termini C4-type zinc finger domain (CF), CCHC-type Zinc-Finger domain (ZF), Basic Helix-Loop-Helix domain (bHLH), Lin11, Isl-1, and Mec-3 domain (LIM). For other designations, see **Figure 1**.

The β-globin locus was the first gene cluster at which long-range (about 40 kb) chromosomal interactions between a distal enhancer, the locus control region (LCR), and the target β-globin promoters during erythropoiesis were described (Carter et al., 2002; Tolhuis et al., 2002). Transcription factor GATA1 was shown to be essential for the induction of most, if not all, erythroid genes (Welch et al., 2004; Fujiwara et al., 2009). The GATA1 protein contains a highly conserved Cys4-type dual zinc finger module (**Figure 4**), with the zinc fingers located closer to the N- and C-termini being named NF and CF, respectively. The CF is responsible (and sufficient) for high-affinity GATA1 binding to the cognate DNA site (WGATAR); NF is also involved in stabilizing GATA1 binding to DNA, but its main function is to interact with different transcriptional cofactors such as FOG (Tsang et al., 1997; Fox et al., 1999), LMO2 (Osada et al., 1997), SP1 (Merika and Orkin, 1995; Gregory et al., 1996; Imanishi et al., 2010), Klf1 (Merika and Orkin, 1995; Gregory et al., 1996), and many others.

Most GATA1-regulated events require its binding to FOG1 (**Figure 4**), a coregulator protein containing nine zinc fingers, five of them with the CCHC arrangement of zinc-chelating residues. Four of the FOG1 protein zinc fingers bind GATA1 with a similar modest affinity *in vitro*, each contributing to the ability of FOG1 to regulate the transcriptional activity of GATA1 (Fox et al., 1999). Thus, a single FOG1 molecule can potentially interact with several GATA1 molecules bound at separate sites. However, previous studies indicate that FOG1 with a single intact GATA1-binding zinc finger is sufficient for erythroid differentiation (Cantor and Orkin, 2002). Therefore, simultaneous binding of many GATA1 molecules appears to be an excess function of FOG1. As a consequence of their interaction, FOG1 and GATA1 mutually facilitate each other’s binding to chromatin and, in particular, to the β-globin gene promoter (Mancini et al., 2012).

TAL1 and E2A (**Figure 4**) are members of the basic helix-loop-helix (bHLH) family of transcription factors (for review, see Anantharaman et al., 2011). TAL1 heterodimerizes with E2A and binds to canonical DNA sequences, CANNTG, termed E-boxes, each monomer recognizing one-half of the E-box (Massari and Murre, 2000). Many other HLH proteins can also interact with E-box elements in erythroid cell-specific genes, with the specificity of these interactions being in particular determined by nearby bound transcription factors. Tal1 is among the earliest expressed transcription factors important for the specification of hematopoietic cells. Tal1 exists as part of different activator and repressor complexes and is responsible for the activity of many proteins activated during erythropoiesis. Genome-wide analysis of protein-DNA interactions has shown that Tal1 can be recruited to DNA either directly via E-box or in a DNA-binding-independent manner, through interaction with other transcription factors (Kassouf et al., 2010). Dissection of the TAL1-E2A interface shows weak interaction with DNA, suggesting that the complex can bind regulatory regions in cooperation with additional DNA-bound transcription factors (El Omari et al., 2013).

**FIGURE 5:**
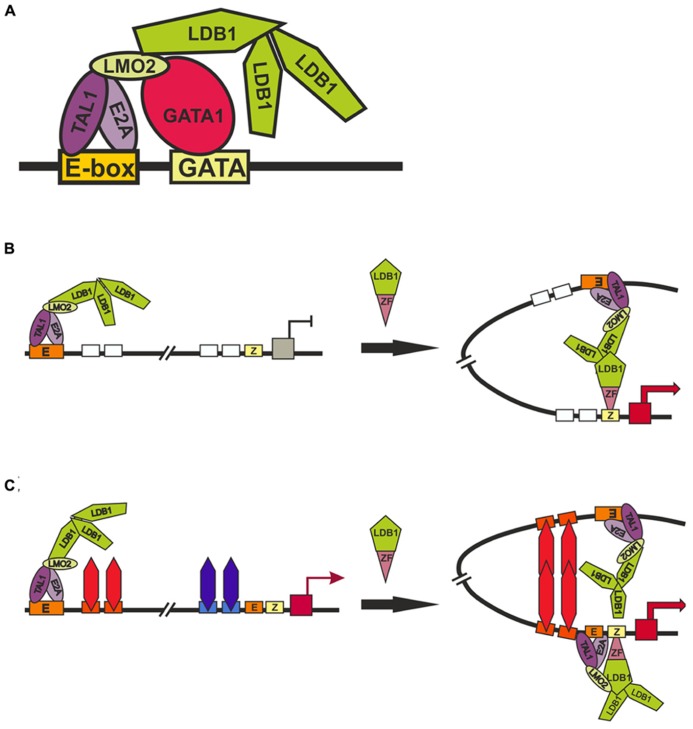
**Models of long-distance interaction between LCT and the β-globin promoter.** **(A)** Scheme for the formation of the Ldb1 complex at the E-box and GATA-sites. **(B)** and **(C)** two alternative models describing role of Ldb1 in organization of distance interactions. Designations: E-box (E), GATA1-binding site (GATA), zinc-finger binding site (Z).

The TAL1:E2A heterodimer interacts with the LMO2 protein and its partner, LDB1 (LIM domain -binding protein 1; Lécuyer and Hoang, 2004). LMO2 (**Figure 4**) is a versatile adaptor protein that, through interaction with additional regulators, plays a critical role in recruiting complexes to DNA. LMO2 comprises two LIM domains that act as protein-interaction motifs (Wadman et al., 1997). A single LMO2 molecule bridges the DNA-binding proteins GATA1 and TAL1/E2A, thereby creating a stable complex on DNA (Wadman et al., 1997; Wilkinson-White et al., 2011; El Omari et al., 2013). The DNA contacts are made by TAL1/E2A heterodimers and the C-terminal zinc-finger of GATA1. The GATA1 NF binds the C-terminal half of the LIM2 domain of LMO2, leaving LIM1 and the N-terminal half of LMO2 available for contact with TAL1/E2A (Wilkinson-White et al., 2011). The Ldb1 protein (**Figure 4**) can interact with multiple transcription factors and mediate interactions between them (Matthews and Visvader, 2003). This protein contains the N-terminal self-association domain that forms trimers (Cross et al., 2010), and its C-terminal domain is involved in the interaction with LMO2. The multiprotein complexes containing GATA1, TAL1, E2A, LMO2, and LDB1 proteins (named Ldb1 complexes) bind to a conserved paired motif composed of a consensus E-box and a GATA motif (**Figure 5A**) with restricted orientation and spacing, CANNTG-N8-10-GATA (Cheng et al., 2009; Soler et al., 2010; Li et al., 2013).

Genome-wide analysis has revealed a high percentage of overlapping binding sites for KLF1 (**Figure 4**) and the Ldb1 complex near TSS or within the first intron at putative erythroid lineage–specific promoters (Tallack et al., 2012; Li et al., 2013). It is supposed that Klf1 and the Ldb1 complex function cooperatively to regulate transcription of shared target genes during erythropoiesis. In particular the major globin promoter and LCR contain a number of EKLF-binding sites (Perkins, 1999; Bieker, 2001). KLF1 recognizes the CACCC-box motif, which is found in erythroid-specific gene promoters and is required for their activation (Yien and Bieker, 2013). KLF1 contains three similar C2H2 zinc fingers at the C-terminus that comprise its DNA-binding domain. KLF1 interacts with components of the basal transcription machinery, such as the p62 subunit of TFIIH (Mas et al., 2011), and with TAF9 (Sengupta et al., 2009). These interactions are necessary for stabilization of transcription machinery on promoters, the β-globin promoter in particular (Sengupta et al., 2009; Mas et al., 2011). GATA1 can physically interact with KLF1 and exhibits functional synergy with KLF1 at erythroid promoters (Merika and Orkin, 1995; Gregory et al., 1996). KLF1 also interacts with chromatin-modifying and remodeling factors, such as P/CAF, CBP/p300, SWI/SNF complex, and possibly BAF47/BAF155 (Yien and Bieker, 2013). Erythroid cells that lack KLF1 exhibit an aberrant chromatin configuration and altered components at KLF1-dependent target promoters, the β-globin promoter in particular, resulting in histone hypoacetylation, loss of DNase I hypersensitivity, and the absence of CBP, BRG1, TBP, and RNA polymerase II (Pol II; Bottardi et al., 2006). Thus, KLF1 is essential for the formation of erythroid-specific active promoters.

Inactivation of GATA1 and its cofactors – FOG1 (Vakoc et al., 2005), KLF1 (Drissen et al., 2004), and Ldb1 (Song et al., 2007) – proved to strongly reduce the expression of β-globin gene and impair interactions between the LCR and promoter. These results were interpreted as evidence for the involvement of these factors in long-distance interactions between the LCR and the promoter. One of the main problems in interpreting the results of experiments on RNAi-mediated inactivation of tested genes is that these transcription factors are of general importance for stimulating transcription of the genes during erythropoiesis and, in addition, are subject to cross-stimulation (Tallack et al., 2010; Mancini et al., 2012; Li et al., 2013). Thus, the inactivation of any of the factors may lead to changes in the expression of other known and unknown factors that are involved in the distant enhancer–promoter interactions. To overcome such a problem, an elegant model system has been developed that employs artificial zinc fingers to tether Ldb1 to the β-globin promoter in GATA1-null erythroblasts (G1E cells), in which the β-globin locus is inactive (Deng et al., 2012). Since G1E cells lack GATA1, the β-globin promoter is devoid of Ldb1, whereas the LCR retains its activity and ability to bind Ldb1. Experiments with this model has shown that the targeting of Ldb1 or its self-association domain alone to the β-globin promoter substantially activates transcription in the absence of GATA1 and that promoter tethering of Ldb1 provides for the formation of a 40-kb chromatin loop between the LCR and promoter and for transcription activation. According to the authors, their findings support the model that the self-association domain of Ldb1 is an essential rate-limiting effector of GATA1 during chromatin loop formation between the LCR and promoter (**Figure 5B**). However, since the self-association domain of Ldb1 is relatively weak (Cross et al., 2010), it is difficult to imagine how the binding of one Ldb1-ZF chimeric protein to a single site in the promoter region of the β-globin gene can provide for the establishment of specific interaction with the LCR located at a distance of 40 kb. Indeed, the authors themselves have shown that nonspecific dimerization domains of GAF, lexA, and p65NFkB proteins recruited to the LCR and promoter fail to support this distant interaction (Deng et al., 2012).

Alternatively (**Figure 5C**), the chimeric Ldb1-ZF protein can possibly substitute for GATA1 by facilitating the recruitment of E2A/TAL1/LMO2 complex to the promoter. This explains why the recruitment of the LMO2-interacting domain of Ldb1 alone proved to be sufficient for partially restoring the expression of β-globin gene and the distant interaction of the promoter with the LCR (Deng et al., 2012). In this case, we assume the existence of some unknown architectural proteins that bind to the LCR and promoter region and support distant interaction between them only when the promoter is active. This model explains the role played in chromatin loop formation by Brg1, the ATPase component of the SWI/SNF nucleosome remodeling complex (Kim et al., 2009), the general transcription factor TFII-I (Ren et al., 2011), and transcription factors such as KLF1, FOG-1, and GATA1 (Drissen et al., 2004; Vakoc et al., 2005, Song et al., 2007). All these factors are required for the formation of active β-globin promoter, with consequent activation of putative architectural proteins that form the chromatin loop with the LCR. In addition to organizing specific distant interactions, these architectural proteins must remain on the regulatory elements during mitosis. In contrast to most other DNA-binding factors, GATA1 remains bound to the subset of its target genes during mitosis (Kadauke et al., 2012). All examined GATA1 cofactors (FOG1, TAL1, Ldb1, and LMO2) vacate mitotic chromatin regardless of whether GATA1 is retained, which indicates that they do not influence GATA1 binding to mitotic chromatin. However, inactivation of GATA1 only partially affects DNaseI hypersensitivity (HS), suggesting that additional unknown factors are involved in the formation of nucleosome-free regions. HS propagation through mitosis is also mediated by a GATA1-independent mechanism. These findings may be regarded as evidence for the existence of not yet identified architectural proteins that form a mitotically stable platform for the binding of GATA1 and reassembly of coregulator complexes at the appropriate genomic locations.

There is ample evidence for possible involvement of several other proteins in organization of long-range interactions, including the transcription factor SP1 that contains C2H2-type zinc finger DNA-binding domain and glutamine-rich dimerization domain (Courey et al., 1989; Mastrangelo et al., 1991; Su et al., 1991), the transcription factor Klf4 (Wei et al., 2013) that interact with many transcription regulators, including Oct4 and Sox2 (Wei et al., 2009), general activator p300/CBP, and repressors such as HDAC and CtBP (Swamynathan, 2010), MAR-binding protein SATB1 (Cai et al., 2006; Gong et al., 2011), TFIIIC (Kirkland et al., 2013), and condensins (D’Ambrosio et al., 2008). In any case however, it would be premature to arrive at any definitive conclusions about the role of these proteins in the chromosome architecture.

## CONCLUSION AND PROSPECTS FOR THE FUTURE

Chromatin looping between different types of regulatory elements (promoters, enhancers, silencers, and insulators) is widely observed and appears to be a general mechanism for establishing long-range functional interactions in the genomes of higher eukaryotes. In contrast, distant interactions between regulatory elements in yeast are relatively rare. For example, GAL4 activator can stimulate yeast promoters only when its binding sites are located in relatively close proximity to the promoter, at a distance of no more than a few hundred base pairs (Guarente and Hoar, 1984; Struhl, 1984). Thus, we can postulate that higher eukaryotes possess a special class of architectural proteins responsible for distance interactions, which are absent in the yeast genome. It is important to note that the cohesin and Mediator complexes are highly conserved among all eukaryotes (Ansari and Morse, 2013). In yeast, the Mediator complex is recruited to GAL4 activator sites (Reeves and Hahn, 2005; Ansari and Morse, 2013). Moreover, cohesin is likely to influence transcription in *Saccharomyces cerevisiae* via interaction with the Mediator complex (Cena et al., 2013). If cohesin and Mediator can support interactions over distances of many kilobases in mammals, it is difficult to explain why homologous proteins in yeast fail to stimulate promoter from the GAL4 activator bound at a distance of only 400–500 bp.

Insulator proteins such as CTCF, Su(Hw), and Zw5 have no homologs in the yeast genome, which makes them probable candidates for organizing distant interactions. To consistently support such interactions in chromosomes, putative architectural proteins should be able to remain bound to chromosomes during mitosis, the process that imposes dramatic and dynamic changes on nuclear organization (Kadauke and Blobel, 2013). In contrast to most transcription factors, the Su(Hw) and dCTCF proteins in *Drosophila* and CTCF in mammals have predominantly constitutive binding sites in different cell lines and tissues (Chen et al., 2008; Song et al., 2011; Lee et al., 2012; Schwartz et al., 2012; Soshnev et al., 2012, 2013; Wang et al., 2012), suggesting that these transcription factors bind to chromosomes during the cell cycle. Contradictory results concerning the potential binding of CTCF to mitotic chromosomes (Burke et al., 2005; Komura et al., 2007; Wendt et al., 2008) may be explained by difficulties in selecting suitable antibodies for which the recognizable epitope is not occluded due to chromatin compaction during mitosis and mitosis-specific post-translational modifications (Kadauke and Blobel, 2012). Additional experimental approaches are required to elucidate the ability of the insulator proteins to bind to their sites through the cell cycle. Another still unresolved question is how architectural (insulator) proteins can organize specific interactions between distantly located sites. It appears that, to this end, they should have special homodimerization domains. If so, the role of cohesin is limited to supporting already established distant interactions.

The important but as yet unresolved question is as to how long-distance interactions are regulated. Possible roles of non-coding RNAs and different protein modifications in stimulation/repression of such interactions are discussed in several recent publications (Herold et al., 2012; Lee and Iyer, 2012; Li et al., 2013; Merkenschlager and Odom, 2013).

Hopefully, further studies will provide a deeper insight into the mechanisms of specific long-distance interactions, their regulation, and the principles of organization of chromosomal architecture in higher eukaryotes.

## Conflict of Interest Statement

The authors declare that the research was conducted in the absence of any commercial or financial relationships that could be construed as a potential conflict of interest.
